# From insights to action: Enriching the clinical and translational research workforce by developing diverse and inclusive career programs

**DOI:** 10.1017/cts.2022.434

**Published:** 2022-08-01

**Authors:** Raquel Ruiz, Alfred Vitale, Ann Schwartz, Deborah Ossip, Martin S. Zand, Ann Dozier, Leonard E. Egede

**Affiliations:** 1 University of Rochester Clinical and Translational Science Institute, Rochester, NY, USA; 2 Center for Leading Innovation and Collaboration (CLIC), CTSA Program Coordinating Center, Rochester, NY, USA; 3 Department of Public Health Sciences, University of Rochester, Rochester, NY, USA; 4 Department of Medicine, Division of Nephrology, University of Rochester, Rochester, NY, USA; 5 Division of General Internal Medicine, Medical College of Wisconsin, Milwaukee, WI, USA; 6 Center for Advancing Population Science, Medical College of Wisconsin, Milwaukee, WI, USA

**Keywords:** Diversity, training, mentoring, translational science, workforce, clinical research

## Abstract

Diversification of the Translational Science workforce is a strategic goal for the National Center for the Advancement of Translational Science (NCATS) program. NCATS has identified the development of translational science education, training, and support for a diverse translational science workforce as key to advancing the growing field of translational science. An annual mixed-methods assessment has been conducted on Common Metrics data submitted by over 60 Clinical & Translational Science Awards (CTSA) programs nationwide and includes metrics addressing recruitment and retention of scientists with particular attention to underrepresented persons and women. This article describes a methodology for the development of *From Insights to Action*, a resource for guiding program implementation and strategic planning to develop a diverse clinical and translational science workforce. This was informed by the Common Metrics Initiative process and constituted of findings from qualitative interviews of a subset of CTSAs that participated. The dissemination of this guide had several impacts, including providing structural foci for the CTSA Fall 2020 program meeting centered on Diversity, Equity, and Inclusion in translational science; addressing NCATS’ goal of workforce diversity; and understanding the number of diverse graduates still engaged in research.

## Introduction

Research shows that diverse teams working together and capitalizing on innovative ideas and distinct perspectives outperform homogenous teams [1]. Scientists and trainees from diverse backgrounds and life experiences bring different perspectives, creativity, and individual enterprise to address complex scientific problems [1]. For the National Institutes of Health (NIH)-supported scientific workforce, diversity comes with many tangible benefits, including fostering scientific innovation, enhancing global competitiveness, contributing to robust learning environments, improving the quality of the researchers, advancing the likelihood that underserved or health disparity populations participate in and benefit from health research, and enhancing public trust [1]. However, diversification of the scientific workforce has advanced slowly.

The two primary vehicles for career development in Clinical & Translational Science Awards (CTSAs) at the time of this analysis have been the KL2 Career Development and TL1 training grants (since this analysis, however, National Center for Advancing Translational Science (NCATS) has added K12 and pre-/post-doctoral T32 awards). KL2 awards [2,3] offer formal research training experience for clinical investigators who have recently completed an M.D., Ph.D., or equivalent doctoral degree and who are commencing basic, translational, and/or clinical research. All CTSA Program hubs have a KL2 program, which provides trainees with a rich, mentored career development experience in a multidisciplinary setting. KL2 scholars come from a variety of fields (e.g., medicine, dentistry, nursing, the behavioral sciences, biostatistics, and epidemiology) and typically receive two years of NCATS-funded protected research time and mentored career development support. Many CTSA Program hubs also include TL1 pre- and post-doctoral programs that provide trainees with an introduction to clinical and translational research [4,5]. CTSA hubs recruit TL1 candidates for full-time predoctoral training, combined health-professional doctorate-master’s training, and postdoctoral fellows. The goal of the TL1 program is to increase the number of well-trained clinical and translational scientists who can lead the design and oversight of future clinical investigations critical to the overall mission of NCATS and NIH.

Initiated in 2015 for the NIH-funded NCATS program, the Common Metrics Initiative (CMI) [6] has been implemented in over 60 CTSA programs [7]. A key component of the Common Metrics is the Careers in Clinical and Translational Research metric, which measures the recruitment and retention of scientists with particular attention to underrepresented persons and women [8]. The metric also supports the NCATS strategic goal of developing and fostering innovative translational training of a highly skilled, creative, and diverse translational science workforce [9]. This national goal recognizes that advancing the field of translational science is through translational science education and training that supports a diverse translational science workforce.

This article describes the methodology employed for engaging CTSAs beyond the CMI process to identify and articulate strategies they were implementing to enhance the diversity of scientists within the field of translational science and will outline the findings from the culminating report disseminated across the CTSA consortium, *From Insights to Action.* This report summarizes strategies learned from interviewing these academic institutions and is designed to serve as a resource and to guide program implementation and development of strategic institutional plans.

## Background

The Center for Leading Innovation and Collaboration (CLIC), the coordinating center for the CTSA Programs, was charged with implementing the CMI across the consortium [10]. The *Insights to Inspire* (*I2I*) Program was developed as a way to highlight the innovative and unique strategies implemented by participating CTSA institutions to improve on a given Common Metric [11]. These insights have been disseminated via a series of blogs, webinars, and webcasts. CLIC dedicated its *Insights to Inspire* 2020 series to the Careers in Clinical and Translational Research metric [8], with underrepresented persons (URP) and women as the specific measures of interest. We focused on understanding how TL1 and KL2 programs at 18 participating CTSA hubs successfully implemented diversity, equity, and inclusion (DEI) approaches to the recruitment and retention of these future scientists. The approach used qualitative and quantitative methods specifically designed to gain a deeper understanding of implementation within the larger context of what each academic healthcare institution was doing with regard to diversity. For the 2020 *I2I program*, two separate iterations of qualitative analysis were conducted resulting in different themes and products. In the initial qualitative analysis of the program narratives (Turn-the-Curve Plans), five thematic domains emerged: diversity and inclusion, recruitment, application and screening, mentoring, and follow-up and evaluation. We then extended the analyses of hub successes from *Insights to Inspire* to create “*From Insights to Action*," with the goal of providing a sustainable resource of implementable actionable steps for program and institutional leaders on how to recruit and retain a diverse scientific workforce. It is this secondary qualitative analysis of the hub interview scripts that is the focus of this paper.

## Methods

Annually, CLIC generates a Common Metrics Initiative Multi-Year Report that represents the Common Metric Initiative data values for each of the CTSA Program hubs in a de-identified and aggregate form. The Careers metric was the focus of the qualitative analysis for the annual report released in 2019, which included data from 2015 to 2018. Two approaches were taken to selecting the hubs with the most quantitative improvement among the 64 CTSAs whom submitted 2018 data. First, we looked at the institutions who were in the top 50% of metric improvement. Then we also looked at the top four institutions with the best results for either KL2 and/or TL1 data. The total number of institutions resulting from this were 30/64 CTSAs. The analysis used the Results Based Accountability Framework [12] and the Story Behind the Curve sections of each institution’s Turn-the-Curve (TTC) plans. These sections explain both positive and negative factors that affected institutional data values. The process for annual data reporting, hub selection, and interview process is fully described separately[11].

*Hub Interviews:* Eighteen unique hubs out of the initial 30 hubs selected participated in 19 interviews (one institution was selected for both their KL2 and TL1 programs). The interviewees consisted of principal investigators, program directors, administrators, and/or evaluators. On average, the interviews were between 30 and 45 minutes long. The interviews were conducted primarily by a CLIC Communications specialist and the CMI team. Table 1 shows the semi-structured interview protocol, designed to allow for participants to steer the direction of the conversation to address what they felt was meaningful. During the interviews, hubs were asked to share the strategies, insights, and processes that they believed led to the improvement of their metric values and were probed on the five themes identified from the TTC qualitative analysis.

**Table 1.** Semi-structured hub interview protocol

What specific strategies did you implement between 2017 and 2018? Provide as much detail as possible. Who was on the team? How were the strategies implemented? Were the strategies focused on scholars, faculty, or the institution? What challenges did you face? How did you address them? How do these strategies impact the future of clinical and translational research? What are you next steps for promoting diversity and inclusion at your CTSA? What recommendations do you have for other hubs?

Note: During the interview, the institution was asked about the themes identified from the initial qualitative analysis from the Turn-The-Curve plans. The results of this interview led to these products: 1) blogs and 2) webinars.

CTSA: Clinical &Translational Science Awards

### Commonality Analysis

The CMI team conducted a manual and iterative thematic coding process on the interview scripts to identify potential themes for the recruitment and retention of the scientific workforce. An initial round of concept coding examined larger units of text and quotes for general ideas/concepts prevalent in the data. A subsequent round of focused coding categorized the initial concepts as salient potential themes for discussion. These final themes were selected based on categorical overlap and consolidated into more comprehensive themes. The goal was not to provide answers or solutions to other hubs but to assist them in their efforts to identify areas of improvement at their own institutions – solutions that meet the needs of their trainees and scholars.

## Results

**Fig. 1.**
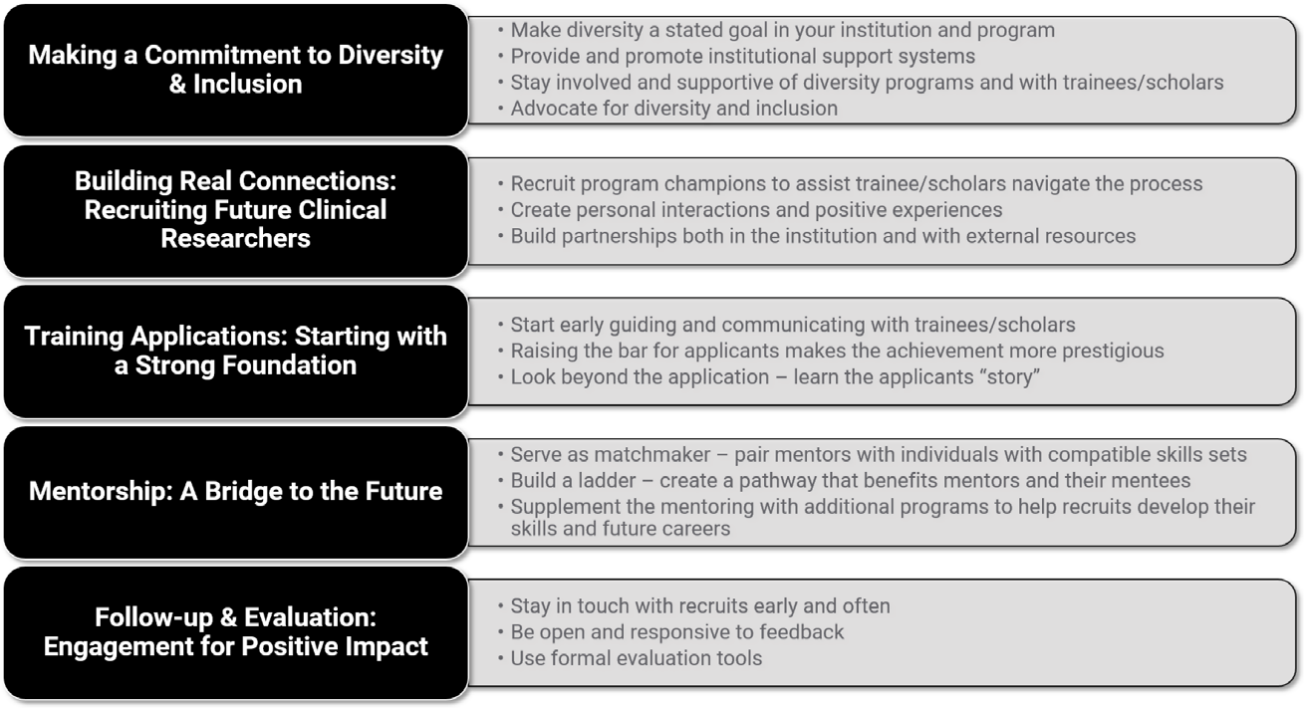
Five themes from interviews translated into blogs and webinars.

*Initial Analysis*: The information obtained in the interviews resulted in a series of blogs and webinars in which selected institutions highlighted effective strategies related within one of the themes (Fig. 1).

The blogs added relatable dimensions to the process, specifically the key messages that could resonate with the CTSA audience, and empowering participants to share their wisdom and expertise among their colleagues and amplify important DEI strategies to a wider audience than the individual hub. The themes published in the blogs and in the *From Insights to Action* resource were reviewed by all participating institutions prior to dissemination. In qualitative research, this process is referred to as member-checking: when data, analytic categories, interpretations, and conclusions are tested with members of those groups from whom the data were originally obtained [13]. This consensus step involves sharing the work of others (e.g., the survey results, interviews) and attributing quotes to institutions, through which credibility with the community is established [13].

*Secondary Analysis:* The secondary analysis of 667 minutes of interview recordings resulted in the six themes covering diversity and inclusion efforts at the participating hubs, outlined below. The themes were translated in the *From Insights to Action* resource into six primary sections, each representative of a strategy identified through qualitative analysis of the hubs interview transcripts. The strategies are not presented in order of importance or merit; readers are encouraged to browse each in the order of their choosing. The sections are composed of brief thematic overviews and a series of discussion questions designed to spark conversation around that theme. The overarching “guiding question” introduces the series of group questions to guide a team conversation. Answers to these questions are not provided as these will be specific to the local context of the program and/or institution. Table 2 provides a summary of the findings along with these conversation-starter questions. Below are the six themes identified from the secondary analysis:

**Table 2.** Emergent themes / key findings from secondary analysis

**PRIORITIZING REPRESENTATION** Prioritize representation, holistically, at every stage of the career pathway.	
**Description:** Diversity and inclusion within the Clinical & Translational Science Awards (CTSA) Program is reflected in many forms: race, ethnicity, gender, area of study, stage of career development, and more. Hubs consistently noted that finding intentional, sustainable, scalable, and public means to represent a wide variety of perspectives in every stage of their programming was a key contributor to the creation of a welcoming and empowering environment. It matters who your scholars and trainees see and hear when they are recruited for, apply for, participate in, and work with your programs. “Matters” refers to the extent to which their race/ethnicity, gender, discipline, etc. are reflected throughout the process.	
**Strategic Question:** In what ways are diverse perspectives represented/supported/encouraged in your programs in each of the following areas?	
**Sub-themes**	**Guiding Questions**
**Programmatic leadership**	From what departments and cultural backgrounds do program Principal Investigator’s (PIs) represent? In what ways can program alumni take on leadership roles? In what ways do program leaders serve as relatable role models for prospective scholars and trainees?
**Review and selection of committee members**	Whose voices are included on the committee in charge of selecting your scholars and trainees? What departments and backgrounds do they reflect? Is there an appropriate space to include a diversity and inclusion advocate and/or a community member? What do you want your scholar or trainee community to look like? Is your committee reflective of these goals?
**Accessible mentors and role models**	How do mentors become involved with the program? From what departments and backgrounds are they recruited? How are faculty selected or recruited to become mentors? In what ways are alumni encouraged to continue their engagement with the program as role models and mentors to future scholars and trainees? Are there pathways for this transition?
**Accessible connections to the local community**	In what ways can scholars and trainees engage with community members? Are there formal structures, like community liaisons, to facilitate these connections? In what ways are the needs and voices of the community represented in the program?
**Representatives speaking at programmatic events**	In what ways can program alumni speak with current scholars and trainees about their experiences? What types of guests are invited to workshops and speaking events? What disciplines and backgrounds do they represent?
**Inclusive research and faculty initiatives, from early to late-stage**	To what extent does your program participate in early “pathway” initiatives? Are investigators encouraged to pursue diversity supplements for their existing grants? What programmatic opportunities do scholars and trainees have to advance as faculty in your institution? What institutional supports exist to support faculty as they transition from their first R grant to their next?
**Written materials**	Is your program’s commitment to diversity and representation visible? Where? To whom? In what ways does your grant, website, and program application reflect your commitment to representation?

**BUILDING PARTNERSHIPS** Actively collaborate with departmental, institutional, and across-institutional leadership on common/aligned programmatic goals.	
**Description:** Career development programs should not stand in isolation. Whether recruiting internally for a K program or across state borders for T program, hubs held partnerships in high esteem. From engaging in conversations with the leadership of the medical center about shared diversity and inclusion goals or working with a partner institution to enrich the pool of talented recruits, partnerships can take on many forms. Actively seeking out partners on — and off — your campus can further the representation efforts, bolster program recognition, and create meaningful, long-lived connections and collaborations.	
**Strategic Question:** In what ways do your programs intentionally seek out active partnerships with each of the following entities?	
**Sub-themes**	**Guiding Questions**
**Internal recruitment partners**	What new connections can you make with individuals across departments who can serve as program “champions” or navigators? How will you stay in touch with these designated primary contact points? Do department heads, deans, or administrative leadership recommend students for your programs? How can you communicate the goals and benefits of your program to them? Who is writing letters of support on behalf of your applicants? In what ways can you ask for their help in recruiting an increasingly deep and diverse candidate pool? What connections exist between your program leadership and any diversity and inclusion offices, councils, organizations, or centers on campus? What feedback or shared goals might you pursue?
**External recruitment partners**	Is your institution ready to create formalized collaborations/partnerships with another university? What mutually beneficial goals could arise as a result of such a partnership?
**Advisory boards**	Does your program seek feedback from an internal advisory board? Who is represented in that space? How are they recruited? How frequently do you meet, and in what capacity? In what ways could your program benefit from consultation with diversity advisors for programmatic feedback and recommendations?
**Funders of aligned goals**	Who on campus discusses shared goals regarding diversity, equity, and inclusion? In what ways could you partner to seek institutional funding to support mutually beneficial programs? Are academic enrichment funds available from your institution? Who in administrative leadership do you need to speak with to discuss these types of funding opportunities?
**Community partnerships**	Does your program or center partner with local middle or high schools, teachers, or education professionals? In what ways are young students exposed to research and clinical and translational science as potential career options? What undergraduate research programs does your institution offer? In what ways can your program participate or use diversity and inclusion supplements to increase undergraduate involvement in research? Does your institution or program have community researcher connections or liaisons? How are these community members trained, compensated, and represented within your program?

**DESIGNING PROGRAM STRUCTURE** Balance structured programmatic supports with space for scholar-led innovation.	
**Description:** Hubs enhanced the career development of their scholars and trainees through mentorship, curriculum, fellowship, and unique programming and community engagement opportunities. The variety of program structures that work to support the growth of scholars and trainees provide mutually accountable spaces with clearly communicated expectations and benefits. Scholars’ needs may extend beyond the existing programmatic framework, though. Many hubs have acknowledged and supported their scholars as they built structures themselves: exercise groups, affiliation groups, or informal K-clubs, for example. Across hubs, program leaders ongoing, iterative work to balance built-in program supports with nurtured space for scholar-driven action continues to advance their capacity to identify and respond to the dynamic needs of their scholars.	
**Strategic Question:** In each of the following areas, where does your program rely on clear protocols and procedures, and where does it leave control in the scholars’ hands? How is that difference in structure and organization communicated?	
**Sub-themes**	**Guiding Questions**
**Mentoring**	How are mentors recruited into the program? How are expectations communicated to mentors? Which standard criteria are used to vet prospective mentors? How are mentors trained? What resources or events are accessible to mentors, scholars, and trainees for ongoing growth in mentorship skills? How are mentor-mentee pairs formed? What supports exist to ensure a good “match”? How do mentors and scholars access these supports? Who can a scholar or trainee turn to if they are experiencing problems with their mentor? How are these communication channels made clear to the scholars? How frequently do structured mentorship meetings take place each year? Is program leadership involved in these conversations? Which components of the mentor-mentee relationship need to be revisited regularly with and without the mentor present?
**Training and Career Development**	What structured programs or events exist to support prospective scholars and trainees in their preparation of a program application? How are these structures publicized? To whom? Does the institution require that scholars and trainees draft Individual Development Plans (IDPs)? When do scholars and trainees draft Individual Development Plans (IDPs)? Who helps facilitate this process? What structures are in place for scholars, mentors, and program leaders to revisit and revise these documents? How does the IDP help the scholar plan for post-program opportunities? What resources and events are accessible to scholars and trainees as they write grants for independent funding? Who can scholars talk to about navigating the grant-writing process? How do they know to turn to this person or group of people? How can scholars’ writing be reviewed and evaluated in a low-stakes environment? What structures exist to ensure this review process is respectful and constructive?
**Peer-to-Peer Support**	How are multidisciplinary connections between scholars and trainees facilitated formally? Informally? What opportunities for networking are offered to scholars and trainees? In what contexts do scholars come together for meetings? If topical, how are scholars’ voices incorporated in choosing meeting content? What opportunities exist for scholars to give one another feedback in these settings?
**Recruitment**	Is the program involved in foundational programs offered by the institution that bring high school and undergraduate students into the research space? How are scholars and/or mentors prepared for this involvement? What steps are taken to ensure clarity in application expectations and program benefits to prospective scholars and trainees? Are there designated primary contact points — program “champions” or navigators — that prospective students expect to hear from? How are these individuals identified? How are marketing efforts systematically amplified outside of email channels? How early does formal or informal “recruitment” start? When and how does the program reflect on recruitment strategies and make adjustments for upcoming cycles? What efforts, systems, or formal programs are in place to retain recruits within the institution after the program?
**Application**	Prior to submitting an application, do prospective scholars need to provide a Letter of Intent? How are scholars prepared for these prapplication components, and is it clear how they will be evaluated? How closely does your program’s application and review process mirror that of the National Institute of Health (NIH)? In what ways does your program harness similarities to prepare scholars and trainees for independent grant writing?
**Curriculum**	Are concepts like health disparities and community engagement included in program curricula? Are scholars aware of the demands of the program’s curriculum? Do they know what to expect in terms of commitment and outcomes?
**Feedback**	Where do scholars and trainees go to provide feedback if they have ideas or concerns regarding the program structure? How do they know to turn there? How is this process communicated to scholars and trainees?

**MAKING IT PERSONAL** Value and nurture the whole scholar: past, present, and future.	
**Description:** Scholars and trainees may only be with your program for two years; however, their experiences may serve as catalysts for future careers in clinical and translational science research. Comradery, collegiality, and care were consistently referenced by hubs when asked how they created environments welcoming to scholars of all backgrounds. Hubs described ways in which — through both words and actions — they celebrated scholars’ pasts, welcomed scholars to their career development community, and helped scholars plan for a fulfilling future.	
**Strategic Question:** Where in your programs can scholars turn to meet their holistic needs?	
**Sub-themes**	**Guiding Questions**
**Holistic application**	In what ways are alternate pathways into clinical and translational science research considered in the program’s application? Is there space to discuss adversity or challenges faced? When and where are in-person interviews conducted? Do scholars have the chance to express their interest in a non-written format?
**Mentoring**	How is work-life balance addressed in mentor-mentee relationships? Are mentors prepared to have conversations beyond the scope of the advancement of their mentee’s professional career?
**Networking**	What peer-to-peer support groups exist within or outside of your program that scholars and trainees can turn to?
**Community building**	Are there spaces to gather or celebrate with scholars as a group outside of workshops and formal events?
**Future-forward planning**	How often do you discuss non-outcomes-oriented (publications, grants) topics with scholars and trainees? In what ways do structural program components — like IDPs — map and focus on scholars’ and trainees’ present and future goals?
**Responsive programming**	Through what channels does your program foster bidirectional communication? Do scholars or trainees feel comfortable expressing whether aspects of the program are or are not working for them and their personal-professional schedules? Does the program have a visible track record of responding and changing systems when scholar needs are not being met?

**IMPROVING THROUGH FEEDBACK** Create open, responsive avenues for mutual feedback.	
**Description:** Gathering data from scholars, trainees, and mentors can be an exhausting process. While there is no one fix-all solution for perfecting survey response rates, hubs consistently referenced how actions taken during a scholar’s tenure had lasting reverberations for how and why they continued to engage with the program. Hubs discussed feedback as an ongoing, bidirectional practice that manifests itself in formal and informal ways. From the recruitment phase to alumni follow-up, hubs work to find spaces to solicit, provide, and — importantly — act on feedback not restricted to outcomes or scholarly gains.	
**Strategic Question:** In which of the following areas do your programs actively seek and provide feedback?	
**Sub-themes**	**Guiding Questions**
**Applications and writing**	How closely does your program’s application and review process mirror that of the NIH? In what ways does your program harness similarities to prepare scholars and trainees for independent grant writing? Who receives feedback on their application? How is that feedback delivered? Are those who are not accepted into the program contacted to support future application efforts? Is there time or space to deliver this feedback directly, rather than through email?
**Mentoring and relationships**	Does feedback flow bidirectionally between scholars and mentors? Does the program rely on this feedback cycle to occur organically, or are there structures in place to facilitate the process? How does the program help to foster relationships well suited for the long-term exchange of feedback? What do interactions between scholars and program stakeholders look like after scholars have completed the program? Are there opportunities to check in informally or share opportunities well suited to a scholar’s interests?
**Program implementation**	Who is providing feedback to the program? Are there any stakeholder voices missing from the conversation? How does the program respond to feedback? Who is involved in decision making when changes are made? In what ways is the program “open-minded” when it comes to feedback from scholars, mentors, diversity advisory boards, or the National Center for Advancing Translational Science (NCATS)? When is feedback sought from program alumni? Is there space for long-term retrospection on relative program benefits and gaps?

**WINNING ENDORSEMENT** An inclusive program seeks and sees advocates outside of its own leadership.	
**Description:** Many hubs highlighted their own scholars and trainees as their most valuable recruitment assets. To have program alumni, department heads, and current scholars actively reach out to prospective applicants can be a powerful testament to the work your program has done in creating a welcoming, inclusive environment. Consider who is endorsing your career development program and which voices are missing from the table.	
**Strategic Question:** In what ways does your program create an environment that its participants and partners want to endorse to others?	
**Sub-themes**	**Guiding Questions**
**Scholars, past and present**	What spaces exist for prospective students to speak with program alumni? Which scholars are recommending your program to others? Why? How do you know? What disciplines and backgrounds do they represent? Are there scholars that would not recommend your program? How do you know? And why? Is there an opportunity to rectify?
**Program partners**	Who writes letters of recommendation for your students? What are they expressing as the strengths of the program for their prospective scholar? How can you create a stronger connection with recommenders? What role do department chairs and deans play in recommending students to your program? Why are they enthusiastic supporters — or why not?

1. *Prioritizing Representation*: Diversity and inclusion within the CTSA Program is reflected in many forms: race, ethnicity, gender, area of study, stage of career development, and more. Hubs consistently noted that finding intentional, sustainable, scalable, and public means to represent a wide variety of perspectives in every stage of their programming was a key contributor to the creation of a welcoming and empowering environment. It matters who your scholars and trainees see and hear when they are recruited for, apply for, participate in, and work with your programs. “Matters” refers to the extent to which their race/ethnicity, gender, discipline, etc. are reflected throughout the process.
2. *Building Partnerships*: Career development programs should not stand in isolation. Whether recruiting internally for a K program or across state borders for a T program, hubs held partnerships in high esteem. From engaging in conversations with the leadership of the medical center about shared diversity and inclusion goals or working with a partner institution to enrich the pool of talented recruits, partnerships can take on many forms. Actively seeking out partners on — and off — your campus can further the representation efforts, bolster program recognition, and create meaningful, long-lived connections and collaborations.
3. *Designing Program Structure*: Hubs enhanced the career development of their scholars and trainees through mentorship, curriculum, fellowship, and unique programming and community engagement opportunities. The variety of program structures that work to support the growth of scholars and trainees provide mutually accountable spaces with clearly communicated expectations and benefits. Scholars’ needs may extend beyond the existing programmatic framework, though. Many hubs have acknowledged and supported their scholars as they built structures themselves: exercise groups, affiliation groups, or informal K-clubs, for example. Across hubs, program leaders’ ongoing, iterative work to balance built-in program supports with nurtured space for scholar-driven action continues to advance their capacity to identify and respond to the dynamic needs of their scholars.
4. *Making It Personal*: Scholars and trainees may only be with your program for two years; however, their experiences may serve as catalysts for future careers in clinical and translational research. Camaraderie, collegiality, and care were consistently referenced by hubs when asked how they created environments welcoming to scholars of all backgrounds. Hubs described ways in which — through both words and actions — they celebrated scholars’ pasts, welcomed scholars to their career development community, and helped scholars plan for a fulfilling future.
5. *Improving through Feedback*: Gathering data from scholars, trainees, and mentors can be an exhausting process. While there is no one fix-all solution for optimizing survey response rates, hubs consistently referenced how actions taken during a scholar’s tenure had lasting reverberations for how and why they continued to engage with the program. Hubs discussed feedback as an ongoing, bidirectional practice that manifests itself in formal and informal ways. From the recruitment phase to alumni follow-up, hubs work to find spaces to solicit, provide, and — importantly — act on feedback not restricted to outcomes or scholarly gains.
6. *Winning Endorsement*: Many hubs highlighted their own scholars and trainees as their most valuable recruitment assets. To have program alumni, department heads, and current scholars actively reach out to prospective applicants can be a powerful testament to the work your program has done in creating a welcoming, inclusive environment. Consider who is endorsing your career development program and which voices are missing from the table.

*Reporting:* The above themes framed the report and resource: *From Insights to Action: Enriching the Clinical Research Workforce by Developing Diverse and Inclusive Career Programs* [14]. This resource is designed for institutions seeking insights for developing a synergistic approach for improving their recruitment efforts to diversify the future clinical and translational research workforce. For example, an institution can use the guiding questions in Table 2 as conversation starters for the theme that they wish to improve in their career program. The resource includes all aspects of the lifecycle — from scholar/trainee recruitment through mentorship and follow-up after completion of the program. These insights are based on experiences from the field, from a place of practice.

**Fig. 2.**
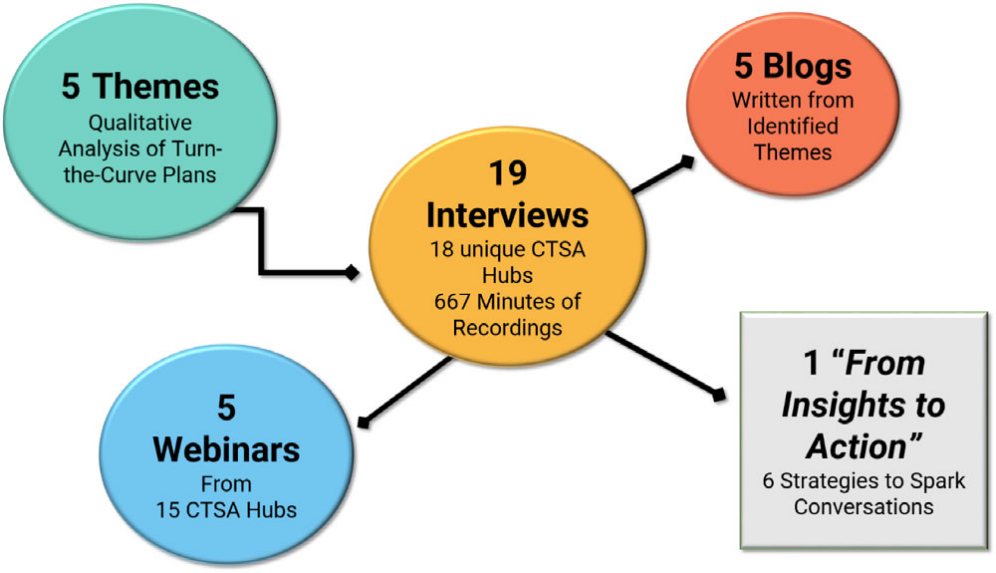
Products from program narratives and interview scripts qualitative analyses (CTSA: Clinical & Translational Science Awards).

Fig. 2 visually summarizes the products of both qualitative analyses conducted.

*Dissemination: From Insights to Action* was disseminated throughout the CTSA consortium via newsletters, the *Insights to Inspire* webpage [15], on key national stakeholder calls, and social media channels. There were 197 unique website page views for *From Insights to Action.* A Twitter campaign began in early 2020 to promote *From Insights to Action* with eight topic-specific tweets to our 849 followers. The number of times (impressions) that these tweets were seen ranged from 163 to 396 across tweets. The Association of American Medical Colleges shared the resource on two of their newsletters on January 2021: Group on Research, Education and Training (GREAT) and Group on Research Advancement and Development (GRAND). Notably, the *From Insights to Action* resource also served as the blueprint for the “Diversity, Equity and Inclusion, Training” breakout session [16] at the CTSA Program Annual meeting in Fall 2020. There were 133 participants in this breakout.

## Discussion

Although the CMI was not perfect, the consortium generated feasible, measurable aggregated metric data from the hubs that served as benchmarks for continued improvement. Through the *I2I* program, specifically for the Careers metric mixed-methods assessment, we identified themes and strategies used by hubs that were most successful in addressing diversity of their KL2 and TL1 workforce/trainees. These were disseminated to the hubs with several impacts:

1. Implemented a consistent approach to measuring trainee diversity at the hub level that allowed for comparisons of median levels at individual hubs and aggregated across the consortium. This addresses NCATS strategic goal of developing a diverse CTS workforce.
2. Highlighted the real impact of the Careers CM by getting hubs to examine the percent of graduates who are “actively engaged in research," the main reason they were trained.
3. Induced hubs to brainstorm new ways to enhance diversity and to disseminate those techniques to the CTSA consortium.
4. Reduced silos by having all hubs participate in the CMI and share best practices (e.g., Insights to Inspire (I2I) Program, From Insights to Action report).

The *From Insights to Action* resource provides a set of strategies addressing various elements to developing a diverse scientific workforce. The data and insights acquired are based on experiences of hubs within the CTSA consortium that showed significant improvement in, or were highest performers on, the associated quantitative common metrics. This resource was disseminated across the CTSA consortium and more broadly to the medical community, with preliminary measure of uptake indicating positive reach.

The *From Insights to Action* report included representation from 18 institutions (fewer than 30%) out of about 64 within the CTSA community. While the CTSA program consortium shares many common programs, and all participate in the CMI process, specific hubs may differ in terms of administrative and institutional structures, community engagement, available resources, and other local factors that may impact the potential for implementing a particular strategy offered in the resource. Further, while it is conceivable that the strategies offered may be generalizable beyond the CTSA program, a comparable inquiry process would need to be conducted across other scientific research programs to better assess them as “best practices.”

### Going Forward

A combination of ongoing, intentional efforts and commitment is necessary to strengthen inclusivity and diversity in career education training programs. It is important for institutional leadership to view this work as a strategic imperative — not just the right thing to do [17]. A purposeful devotion of time and resources for these inclusive initiatives will enable shared goals to flourish. Now with the new addition of the NCATS K12 and T32 programs, further research will be needed to identify unique needs and strategies that account for relevant contextual factors within those programs. Although these ideas represent the work within a unique research community, they are translatable to other programs within and outside of the education field that are in search of starting or improving upon their recruitment and retention of a diverse workforce. Finally, it will be important to evaluate the implementation of these strategies to further understand what works across institutions and programs while accounting for local contextual factors.
